# „Man sitzt in einer Seifenblase, während die anderen Menschen leben.“ – Perspektiven geflüchteter ukrainischer Mütter mit Kindern von 0–3 Jahren

**DOI:** 10.1007/s00103-024-03974-7

**Published:** 2024-11-15

**Authors:** Digo Chakraverty, Anna Neumann, Ilona Renner

**Affiliations:** 1 Nationales Zentrum Frühe Hilfen, Bundeszentrale für gesundheitliche Aufklärung, Köln, Deutschland; 2 Bundeszentrale für gesundheitliche Aufklärung, Maarweg 149–161, 50825 Köln, Deutschland

**Keywords:** Ukraine, Flucht, Interviews, Kleinkinder, Unterstützungsangebote, Ukraine, Refugees, Interviews, Young children, Support services

## Abstract

**Hintergrund:**

Zur Situation von ukrainischen Eltern mit kleinen Kindern, die seit dem Krieg in der Ukraine nach Deutschland geflüchtet sind, ist bislang wenig bekannt. Ziel des Forschungsvorhabens des Nationalen Zentrums Frühe Hilfen (NZFH) ist die Exploration der Perspektive dieser Gruppe auf ihr Leben in Deutschland. In der vorliegenden Analyse wird auf die Belastungen und Ressourcen der Eltern und ihre Kenntnis und Nutzung von Unterstützungsangeboten fokussiert.

**Methoden:**

In 17 ca. einstündigen Interviews wurden von Oktober bis Dezember 2022 aus der Ukraine nach Deutschland geflohene Eltern (16 Mütter, ein Vater) mit Kindern bis zu 3 Jahren befragt und ihre Antworten mittels strukturierender Inhaltsanalyse qualitativ ausgewertet.

**Ergebnisse:**

Als Belastungen beschrieben die Interviewten primär die psychische Belastung durch Krieg und Flucht, negative Emotionen wie Gefühle von Einsamkeit und Niedergeschlagenheit sowie Hindernisse bei der Integration. Ressourcen waren vor allem Netzwerke ukrainisch- und russischsprachiger Menschen in Deutschland sowie persönliche Ressourcen wie Selbstfürsorgekompetenz. Die Vermittlung von Angeboten geschah i. d. R. über elektronische Medien. Als besonders nützlich galten Angebote zur Kinderbetreuung, Sprachkurse und Unterstützung in Gesundheitsfragen. Fehlende Kinderbetreuung war der wichtigste Grund für die Nichtannahme weiterer Angebote.

**Diskussion:**

Die psychischen Belastungen der Befragten indizieren einen Bedarf an möglichst muttersprachlichen Versorgungsangeboten. Kinderbetreuungsplätze können ukrainischen Eltern den Besuch integrationsförderlicher Sprachkurse ermöglichen. Die selbstorganisierten Netzwerke der ukrainisch- und russischsprachigen Community sollten bei der Entwicklung von Interventionen von Beginn an partizipativ eingebunden werden.

## Abstract (en)

**Background:**

Little is known about the situation of Ukrainian parents with children up to the age of three who have fled to Germany since Russia’s attack on Ukraine. The aim of the National Center for Early Intervention research project is to explore the perspective of this group on their lives in Germany. This analysis focuses on the burdens and resources of the parents and their knowledge and use of support services.

**Methods:**

In 17 interviews (duration approx. one hour each), parents (16 mothers, one father) who had fled from Ukraine to Germany with children up to the age of three were interviewed between October and December 2022, and their answers were qualitatively analyzed using structured content analysis.

**Results:**

The interviewees primarily described the psychological stress caused by war and flight, negative emotions such as feelings of loneliness, sadness and lack of motivation, and obstacles to integration as burdens. Resources were primarily networks of Ukrainian- and Russian-speaking people in Germany as well as personal resources such as self-care skills. Supporting services were generally communicated via electronic media. Services for childcare, language courses, and support with health issues were considered particularly useful. A lack of childcare was the most important reason for not taking up other services.

**Discussion:**

The psychological stress of the interviewees indicates a need for therapy capacities in their native language. Childcare may enable Ukrainians to attend language courses that promote integration. The self-organized networks of the Ukrainian- and Russian-speaking community should be involved in the development of interventions for this target group in a participatory manner from the outset.

## Hintergrund

Seit dem Beginn des russischen Angriffskriegs auf die Ukraine am 24.02.2022 sind außerordentlich viele ukrainische Familien nach Deutschland geflüchtet [1]. Zum 30.11.2022 hatte sich die Anzahl ukrainischer Staatsbürgerinnen und Staatsbürger in Deutschland im Vergleich zum Februar des gleichen Jahres auf über 1 Mio. nahezu versiebenfacht [1]. Den größten Anteil der Zugezogenen bilden Menschen unter 18 Jahren; bei den Erwachsenen unter 60 Jahren handelt es sich überwiegend um Frauen mit einem vergleichsweise hohen formalen Bildungsniveau [2, 3]. Aufgrund der EU-Massenzustrom-Richtlinie (Richtlinie 2001/55/EG) konnten Ukrainerinnen und Ukrainer ohne Asylverfahren vorübergehenden Schutz in der EU erhalten [3]. Sie hatten umgehend Anspruch auf Leistungen nach dem Sozialgesetzbuch und vollständigen Zugang zum Gesundheitssystem [4]. Damit unterscheidet sich ihre Situation grundlegend von der Situation anderer Migrantinnen und Migranten (Asylbewerberinnen und -bewerber, Geflüchtete aus anderen Regionen). Während die Integration schulpflichtiger Kinder aus der Ukraine vielfach thematisiert wird [5, 6] und erste Zahlen auf ein beeinträchtigtes Wohlbefinden der 3‑ bis 18-jährigen ukrainischen Kinder hinweisen [3], ist zur Situation ukrainischer Eltern mit Kindern bis zum Alter von 3 Jahren bislang wenig bekannt. Somit erschien eine explorative Untersuchung der subjektiven Lebenswelt der Betroffenen angezeigt [7] und wurde vom Nationalen Zentrum Frühe Hilfen (NZFH) durchgeführt.

Erste Untersuchungen zu ukrainischen Geflüchteten indizieren bereits möglichen Unterstützungsbedarf ukrainischer Mütter mit jüngeren Kindern. So kommen Brücker et al. (2023) zu dem Schluss, dass der Erwerb der deutschen Sprache durch Mütter mit Kindern unter 6 Jahren im Vergleich zu Geflüchteten ohne oder solchen mit älteren Kindern aufgrund mangelnder Betreuungsmöglichkeiten während eines Sprachkursbesuchs verzögert stattfindet [2].

Die vorliegende Studie wurde als Erweiterung der Repräsentativbefragung „Kinder in Deutschland 0-3“ (KiD 0–3) im Jahr 2022 [8] durchgeführt und aus Mitteln der Bundesstiftung Frühe Hilfen des Bundesministeriums für Familie, Frauen, Senioren und Jugend (BMFSFJ) finanziert. Ziel der Erweiterung war die Exploration der Situation von geflüchteten ukrainischen Müttern mit Kindern bis zum Alter von 3 Jahren unter verschiedenen Gesichtspunkten, z. B.:

- Wie erleben die Befragten das Leben mit Kind in Deutschland hinsichtlich der Eltern-Kind-Beziehung, der Erziehung und der Alltagsgestaltung?
- Welche Netzwerke zur Unterstützung nutzen die Familien?
- Welche Unterstützungsangebote für Familien mit kleinen Kindern sind den Befragten bekannt, welche werden genutzt, welche nicht und aus welchen Gründen?

Im Mittelpunkt der vorliegenden Auswertung stehen die Belastungen und Ressourcen der Mütter, ihr Unterstützungsbedarf sowie die Nutzung vorhandener Unterstützungsangebote. Für die Bewältigung der Herausforderungen, die das Leben in Deutschland an die Befragten stellt, ist das Ausmaß an empfundener, tatsächlicher und potenzieller sozialer Unterstützung dabei von großer Bedeutung [9]. Nicht im Fokus dieser Analyse stehen dagegen psychologische Traumata. Dies liegt darin begründet, dass in der zum Zeitpunkt der Erhebung bestehenden Orientierungssituation der erst wenige Monate in Deutschland lebenden Teilnehmenden aktuelle, lebenspraktische Fragen vordringlicher erschienen. Ziel der Untersuchung war, den Umgang mit diesen Fragen mithilfe persönlicher Interviews zu explorieren.

## Methoden

Persönlich geführte Tiefeninterviews können den Interviewten die Gelegenheit geben, in vertraulicher Atmosphäre ihre Perspektiven zu schildern [10]. Für einen geordneten Ablauf der Gespräche, der gleichzeitig Freiraum für eigene Anliegen der Interviewten bietet, haben sich leitfadengestützte, halbstrukturierte Interviews bewährt [10]. Geflüchtete und andere Personen mit geringen Deutschkenntnissen bedürfen dazu besonderer Ansprache, damit sie für die Teilnahme an wissenschaftlichen Studien gewonnen werden können, wie ihre Unterrepräsentation in verschiedenen großen Befragungen zeigt [11, 12]. Besonders Erfolg versprechend sind die Ansprache und Befragung durch Personen aus der gleichen Herkunftsgruppe in der gemeinsamen Muttersprache [13]. Für die Durchführung der Interviews wurde daher über das vom NZFH beauftragte Forschungsinstitut House of Research (HoR)^1^ in Berlin eine ukrainisch-russische Muttersprachlerin mit Erfahrung in der Durchführung von qualitativen Interviews eingesetzt.

### Leitfaden.

Der Interviewleitfaden wurde von den Autorinnen und dem Autor entwickelt. Die Interviews begannen mit einer kurzen Wiederholung des Interviewzwecks und einer allgemeinen Einstiegsfrage („Zu Beginn würde ich Sie gerne etwas näher kennenlernen. Bitte erzählen Sie mir doch etwas zu Ihrer Person und zu Ihrem Kind …“). Dieser Teil des Interviews sollte dazu dienen, eine angenehme und vertrauensvolle Gesprächsatmosphäre aufzubauen und die Gesprächsperson (GP) näher kennenzulernen [14]. Im weiteren Verlauf stand das Interesse am informativen Inhalt der Interviews im Vordergrund, weshalb der Leitfaden eine Reihe von Fragen enthielt, die gestellt werden sollten, wenn die Interviewten auf die jeweiligen Themen nicht von alleine zu sprechen kamen [14]. Der Leitfaden enthielt keine dezidierten Fragen zu potenziell traumatischen Erlebnissen, wohl aber die Anweisung, diesen Themen Raum zu geben, wenn die Interviewten darüber sprechen wollten. Er wurde von einem Übersetzungsbüro ins Ukrainische und Russische übersetzt. HoR überprüfte im Rahmen eines Pretests die Eignung der Interviewerin und testete den Leitfaden, der anschließend von den Autorinnen und dem Autor entsprechend den Rückmeldungen der Interviewerin leicht angepasst wurde.

### Rekrutierung.

Neuere Arbeiten zeigen, dass sich ab einer Anzahl von 12 Interviews i. d. R. ein Grad der theoretischen Sättigung einstellt, der bei einer relativ homogenen Gruppe von Befragten (wie im vorliegenden Fall) aussagekräftige Ergebnisse hervorbringt [15, 16]. Angestrebt wurde daher ein Sample von maximal *N* = 20 Interviewpartnerinnen. Die Rekrutierung der Teilnehmenden erfolgte primär über kooperierende Einrichtungen (Flüchtlingsunterkünfte, pädiatrische Praxen mit Mitarbeitenden mit ukrainischen oder russischen Sprachkenntnissen, Kitas etc.). In die Studie eingeschlossen wurden Eltern, die nach dem 24.02.2022 mit mindestens einem eigenen Kind bis zum Alter von 3 Jahren aus der Ukraine nach Deutschland geflohen waren und Ukrainisch oder Russisch sprachen.

Aus forschungspraktischen Gründen wurden alle Interviews in Berlin durchgeführt. Berlin war seit dem Kriegsbeginn ein wichtiger Ankunftsort für Geflüchtete aus der Ukraine [1]. Die Interviews fanden zwischen Oktober und Dezember 2022 wahlweise in den Räumen des Forschungsinstituts oder an einem von den Teilnehmenden bestimmten Ort in Berlin statt. Sie erhielten eine zu unterschreibende Einwilligungserklärung (wahlweise auf Ukrainisch oder auf Russisch) und im Anschluss eine Aufwandsentschädigung von 60 € in bar (ggf. zuzüglich 20 € Fahrgeld). Die Studiendurchführenden haben vor dem Beginn der Datenerhebung eine forschungsethische Selbstverpflichtungserklärung zum Schutz der befragten Personen unterschrieben. Sie verpflichteten sich beispielsweise dazu, besonders sensible Themenbereiche vorab anzukündigen und das jeweilige Thema nur bei expliziter Zustimmung aufzugreifen.

Die Interviews wurden je nach Wunsch der Teilnehmenden entweder in russischer oder ukrainischer Sprache geführt, mithilfe eines Diktiergeräts aufgezeichnet und durch einen Transkriptionsservice verschriftlicht. Anschließend wurden die Transkripte von der Interviewerin anonymisiert. Die Übersetzung ins Deutsche geschah durch eine KI-Übersetzungssoftware mit Prüfung und Korrektur durch übersetzungserfahrene Muttersprachlerinnen (Ukrainisch/Russisch) und abschließender Abnahme durch die Interviewerin.

### Auswertungsmethode.

Die Transkripte wurden mithilfe der Software MAXQDA (Version 22.7.0; VERBI – Software. Consult. Sozialforschung. GmbH, Berlin, Deutschland) [17] einer strukturierenden Inhaltsanalyse unterzogen. Dabei folgte das Forschungsteam dem deduktiv-induktiven Verfahren nach Kuckartz [18]. Ein Autor und eine Autorin (DC, AN) erstellten anhand der Forschungsfragen ein erstes Kategoriengerüst. Hierbei wurde vor der Auswertung ein Satz an deduktiven A‑priori-Kategorien [18] erstellt, die sich aus den Forschungsfragen ableiteten, z. B. „Ressourcen“. Anschließend codierten DC und AN unabhängig voneinander je 3 Transkripte und konsolidierten das im Zuge der Codierungen erweiterte Kategoriensystem. Durch die Zusammenfassung inhaltlich ähnlicher Äußerungen unter gemeinsame Begriffe erfolgte dabei die induktive Bildung von Kategorien am Material [18]; diese Kategorien wurden dann entweder anderen Kategorien hierarchisch untergeordnet (z. B. die induktive Subkategorie „Netzwerk und Unterstützung“ der deduktiven Kategorie „Ressourcen“) oder bildeten eigene Hauptkategorien. Nach dem dritten Konsolidierungslauf erschien das Verständnis der Kategorien und deren Anwendung so weit harmonisiert, dass für die übrigen Interviews jeweils DC oder AN die Erstcodierung vornahmen, die dann von dem bzw. der jeweils anderen geprüft wurde. Unstimmigkeiten wurden diskursiv gelöst.

### Teilnehmende.

Insgesamt *N* = 17 ukrainische Elternteile (16 weiblich, 1 männlich) nahmen an der Studie teil. Die folgenden Auswertungen stützen sich überwiegend auf Angaben der Mütter. Elf der Interviews wurden auf Ukrainisch, 4 auf Russisch und 2 in beiden Sprachen geführt, mit einer Durchschnittsdauer von 65 min. Die Teilnehmenden hatten ein Kind (*n* = 4), 2 (*n* = 9) oder 3 (*n* = 4) Kinder. Acht Befragte lebten ohne weitere Begleitung in Deutschland, 9 mit Partnerin bzw. Partner oder anderen Verwandten. Zwölf der Interviewten waren bereits im März nach Deutschland geflohen, 4 im April und eine im August 2022. Die Teilnehmenden kamen sämtlich aus östlich von Kiew gelegenen Orten oder aus Kiew selbst und lebten bis auf eine in Süddeutschland wohnende Mutter in Berlin.

### Kategoriensystem.

Die Einteilung der Äußerungen in den Interviews in die a priori festgelegten Bereiche Belastungen, Ressourcen, Unterstützungsbedarf und Nutzung von Hilfsangeboten ergab ein Kategorienschema mit deduktiven Hauptkategorien und diesen zugeordneten, induktiven Subkategorien und ebenfalls induktiv aus den Transkripten ermittelten Aspekten. Die Kategorien sind in Tab. 1 mit entsprechenden Ankerzitaten aufgeführt.

**Tab. 1:** Deduktive Hauptkategorien, induktive Subkategorien und Ankerzitate aus der inhaltsanalytischen Auswertung der Interviews

Hauptkategorie	Subkategorie	Ankerzitat
Belastungen	Kriegs- und Fluchterlebnisse	„Du rennst, sie schießen auf das Auto vor dir, und all das vor deinen Augen. Als wir dort herauskamen, hatten die Kinder ein Fieber von zweiundvierzig Grad. Es war bereits der sechzehnte Tag. Sie brannten bereits. Und wir hatten von den ersten Tagen an eine komplette Okkupation. Es gab keine Medikamente. Überall standen Panzer, überall war Militär, alle Geschäfte waren geschlossen“ (Larysa)
	Belastende Emotionen	„Und dann war der psychologische Zustand mit der Tatsache verbunden, dass man hier allein ist, dass man einsam ist. Und dass du mit dem Kind allein bist“ (Ana)
	Schwierigkeiten mit dem Leben in Deutschland	„Es ist schwierig, etwas zu erreichen, das ist wahr. Wir dachten, in der Ukraine gäbe es Bürokratie … Nein, hier gibt es Bürokratie!“ (Marija)
Ressourcen	Netzwerk und Unterstützung	„Ich hatte unmittelbare Familienmitglieder, die Nachbarn in mein Leben brachten. Es haben sich diese Nachbarn gefunden, und sie brachten mehr Menschen aus Russland und der Ukraine in mein Leben, die seit vielen Jahren hier leben und den Ukrainern sehr helfen wollen. Ich habe auch einen engen Freund, der bei der Caritas arbeitet. Dieses Netz ist in meinem Leben sehr dicht“ (Polina)
	Innere Ressourcen	„Im Allgemeinen kommuniziere ich sehr gerne. Das heißt, ich werde nicht zu Hause sitzen und weinen. Nein! Ich werde also nach Ärzten und Übersetzern suchen. Ich werde den Kindern helfen!“ (Milana)
Nutzung von Angeboten	Vermittlung und Zugang	„Telegram hat eine Gruppe für Mütter in Berlin und eine für Hamburg und eine für ganz Deutschland … bei Telegram gibt es Hebammen in diesem Chatroom, sogar mehrere, die speziell Ratschläge zur Gesundheit, zur Kinderbetreuung oder zu irgendwelchen Problemen geben“ (Angelina)
	Nutzung und Bewertung	„Ja, wir sind umgezogen, aber wir haben den Kindergarten trotzdem nicht gewechselt, weil wir den Kindergarten sehr gerne mögen. Wir mögen die Erzieherinnen sehr gerne. Ich sehe, wie sehr M. sie liebt, wie er morgens losrennt, um sie zu umarmen. Das ist für mich sehr viel wert“ (Larysa)
	Gründe für Nichtinanspruchnahme	„Wenn das Kind in den Kindergarten gehen würde, könnte ich einen Sprachkurs besuchen, psychologische Hilfe in Anspruch nehmen und mich ein wenig um mich selbst kümmern“ (Elizaveta)

Hinweis: Vornamen wurden geändert

## Ergebnisse

Die realen Vornamen der im Folgenden zitierten Personen wurden durch zufällig gewählte, in der Ukraine gängige Vornamen ersetzt, um die Anonymität der Teilnehmenden zu gewährleisten.

### Belastungen

Die Mütter waren einer großen Anzahl und Vielfalt an Belastungen ausgesetzt, wobei retrospektiv genannte Erlebnisse und Zustände durch Krieg und Flucht sowie aktuelle emotionale Belastungen und Schwierigkeiten mit dem Leben in Deutschland im Vordergrund standen.

#### Kriegs- und Fluchterlebnisse.

Die Erlebnisse während des beginnenden Krieges in der Ukraine lagen zum Zeitpunkt der Befragung (Oktober–Dezember 2022) noch kein Jahr zurück und wurden von den Befragten teilweise detailliert geschildert.

Acht der Teilnehmerinnen berichteten von Kriegshandlungen mit Luftalarm, Bombardements und Zerstörung und von ihrer damaligen emotionalen Reaktion darauf. Anfängliche Fassungslosigkeit, wie hier formuliert: „… fängt das alles wirklich an?“ (Solomia), wich den Schrecken durch die militärischen Angriffe, bei manchen gekennzeichnet durch lange Aufenthalte in Bunkern und Verstecken und/oder Leben unter russischer Besatzung. Als prägendes Motiv zieht sich die Angst um die Kinder durch die Erzählungen der Mütter:

„Ich dachte, ich würde verrückt werden. Es könnte nicht schlimmer sein. Vor allem Angst um die Kinder“ (Diana).

Auch nach der Ankunft in Deutschland war die Angst noch präsent, oft als Angst um den Partner oder andere Bezugspersonen:

„Er wird morgen 30 Jahre alt … Er wird in die Nähe der Front geschickt. … jetzt mache ich mir Sorgen um meinen Mann“ (Jeva).

#### Belastende Emotionen.

Abgesehen von den potenziell traumatischen Kriegs- und Fluchterfahrungen sprachen die meisten Mütter auch von aktuell belastenden Emotionen. Einsamkeit war insbesondere bei den ohne erwachsene Begleitung eingereisten Müttern ein Problem, das mit der starken Beschränkung auf die Mutterrolle zusammenhing:

„Und der einzige Grund, warum es jetzt so schlimm ist, ist, dass ich ständig mit dem Kind zusammen bin. Das heißt, es gibt keine Großmutter, keine Verwandten, denen man das Kind übergeben kann. Es gibt keine familiäre Kommunikation, keine Unterstützung durch die Familie. Das heißt, man hat das Gefühl, man sitzt in einer Seifenblase, während die anderen Menschen leben“ (Ana).

Depressive Symptome wie Schlafstörungen, niedergedrückte Stimmung oder Antriebslosigkeit wurden ebenfalls geschildert.

„Ich bin oft schlecht gelaunt. Ich lese die Nachrichten oder ich bin nicht ausgeschlafen, ich fühle mich lustlos, ich kann nicht so viel mit dem Kind machen, wie ich sollte“ (Elizaveta).

#### Schwierigkeiten mit dem Leben in Deutschland.

Das Ankommen und Eingewöhnen in Deutschland war für viele Mütter mit teils großen Schwierigkeiten verbunden. Die Sprachbarriere aufgrund fehlender Deutschkenntnisse der Interviewten war ein sehr häufig genanntes Problem. Sie konnte die Einsamkeit noch verstärken:

„Ich kann keine Freunde finden, ich spreche nicht fließend Deutsch“ (Viktorija).

Auch der beruflichen Integration standen die mangelnden Sprachkenntnisse im Weg:

„Mein großes Hindernis ist, dass ich die Sprache nicht kann. Ich würde überall hingehen. Ich würde jetzt wahrscheinlich auch zur Arbeit gehen“ (Solomia).

Zwölf der interviewten Personen äußerten die Bereitschaft oder konkrete Absicht, die deutsche Sprache zu erlernen oder taten dies bereits (s. Nutzung von Angeboten).

„Ich möchte unbedingt die Sprache lernen gehen. Ich habe so ein großes Unbehagen, wenn ich mich jemandem nicht erklären kann“ (Solomia).

Häufig als problematisch empfunden wurde die Bürokratie und deren Zusammenwirken mit der Sprachbarriere:

„Ehrlich gesagt, dauert alles sehr lange. Und das Problem ist, dass alles in einer anderen Sprache ist“ (Melania).

Bürokratie und ein Mangel an Digitalisierung wurde in Zusammenhang mit der Gesundheitsversorgung beschrieben, aber auch als allgemeiner Eindruck geschildert, oft im Kontrast zum Leben in der Ukraine:

„In der Ukraine haben wir alle Dokumente in unserem Telefon. Und hier steht alles auf Papier, alles in Briefkästen“ (Ana).

### Ressourcen

Die Teilnehmenden erwähnten als Ressourcen vor allem Unterstützungsnetzwerke, bestehend aus Menschen und Institutionen. Neben diesen äußeren wurden auch persönliche Ressourcen wie förderliche Coping-Stile beschrieben.

#### Netzwerk und Unterstützung.

Der größte Teil der von den Interviewten beschriebenen Unterstützung kam von Ukrainerinnen und Ukrainern sowie von Russinnen und Russen, die bereits seit Längerem in Deutschland wohnten, oder von vorhandenen ukrainischen Gemeinden und Institutionen. Die Kontakte bestanden teils schon vor dem Krieg und konnten sich in einem Schneeballeffekt entwickeln. Die Geflüchteten berichteten auch von spontaner Netzwerkbildung innerhalb der Gruppe der seit Anfang 2022 geflohenen Ukrainerinnen und Ukrainer, sodass sie sowohl Hilfe erfuhren als auch anderen helfen konnten: „Und den Neuankömmlingen, die hierherkamen, habe ich bei vielen Fragen geholfen, zum Beispiel beim Ausfüllen der Unterlagen“ (Kristina). Die Hilfe reichte von psychosozialer Unterstützung über die Vermittlung von Wohnraum, administrative und praktische Unterstützung bis zu gegenseitiger Kinderbetreuung. Nahezu alle Interviewten berichteten, von (i. d. R. weiblichen) mitgeflüchteten Familienmitgliedern unterstützt zu werden. Fünf der Interviewten waren mit ihren Partnern nach Deutschland gekommen.

„Mütter, die ohne Familie hier sind, das ist eine ganz andere Situation. Ich kann immer anrufen, die erste Person, die ich anrufe, ist meine Mutter. ‚Mama, hol mich ab. Ma, ich bin in einer Besprechung. Mama, hilf mir mal. Mama, ich schaffe es heute nicht**‘**“ (Polina).

Neben der Hilfe durch andere Ukrainerinnen und Ukrainer stellte auch die Freude am Kind eine Kraftquelle dar:

„Das Baby ist also nicht nur eine große Freude, sondern auch gut für mich“ (Oleksandr).

Die Unterstützung durch andere Personen in Deutschland spielte ebenfalls eine wichtige Rolle, insbesondere, weil einige der Interviewten in deren Wohnungen unterkommen konnten. Dies wurde oft mit dem Ausdruck großer Dankbarkeit erwähnt.

„Die Deutschen, die in Potsdam sind, sind einfach unglaubliche Menschen, die sich engagieren. Es wurde uns gesagt, dass Leute ihr eigenes Geld beigesteuert und ein Auto gekauft haben. Ohne Scheiß!“ (Viktorija).

#### Persönliche Ressourcen.

Viele der interviewten Mütter konnten auf persönliche Ressourcen in Form förderlicher Coping-Stile zurückgreifen: die Fähigkeit, Herausforderungen, wie z. B. Sprachprobleme, aktiv anzugehen oder sich selbst positive Erlebnisse zu verschaffen.

„Ich bin nicht schüchtern. Sei es auch grammatikalisch unkorrekt, was ich sage, aber schweigen werde ich nicht. Ich werde also alles sagen, was ich für nötig halte. Ich gehe fast jeden Tag herum und frage: ‚Wie läuft es? Woran soll ich arbeiten?‘“ (Milana).

„Und jetzt, vor buchstäblich einer Woche, habe ich angefangen, für mich selbst etwas Gutes zu tun … Ich habe mir ein paar Duftkerzen gekauft, ich habe mir eine schöne Tasse gekauft, ich habe mir zum ersten Mal die Nägel machen lassen. Ich habe angefangen, mich nicht für all das zu verurteilen …“ (Diana).

### Nutzung von Angeboten

Die Befragten berichteten von einem breiten Spektrum an Unterstützungsangeboten, die sie bereits nutzten. Sie erwähnten in diesem Zusammenhang viele verschiedene Angebote, die ihnen das Leben in Deutschland und die Integration erleichterten.

#### Vermittlung und Zugang.

Elektronische Kommunikation spielte bei der Information und Vermittlung von Unterstützungsangeboten und Wohnraum eine herausragende Rolle:

„Und als wir ankamen, fanden wir auf Facebook Leute, die schrieben, dass sie bereit waren, eine Familie für die erste Nacht aufzunehmen“ (Viktorija).

Als weitere Vermittlungsinstanzen und gleichzeitig Anbieter von Unterstützung wurden Hilfsorganisationen wie die AWO oder Caritas genannt. Eine wichtige Rolle spielten auch ukrainisch- und russischsprachige Kirchengemeinden.

„Hier in der [Straßenname] gibt es eine russischsprachige Kirche. Ich gehe jetzt in diese Kirche. Es gibt dort einen Anwalt, der uns geholfen hat, weswegen wir hier auch leben. In diesem Haus gibt es sechsundzwanzig Wohnungen, die für Menschen aus dieser Kirche reserviert sind“ (Marija).

#### Nutzung und Bewertung.

Bei der Nutzung von Angeboten standen besonders die Aspekte der Kinderbetreuung, der Überwindung von Sprachbarrieren und Unterstützung in Gesundheitsfragen im Vordergrund. Die Kinderbetreuung, insbesondere in Kita/Kindergarten, wurde von den Müttern sehr positiv bewertet, insbesondere auch, weil die Kinder sich dort wohl zu fühlen schienen. Unterschiede zu den ukrainischen Betreuungseinrichtungen konnten gewöhnungsbedürftig sein:

„Der Monat der Eingewöhnung war sehr schwer … aber so macht man es in Deutschland“ (Polina), dies kann aber ebenso als positiver Kontrast wahrgenommen werden: „… in unseren Kindergärten [in der Ukraine; Anmerkung der Autoren] muss er im Alter von zwei Jahren alle Zahlen und alle Farben kennen. Er sollte alles wissen. Niemand muss hier und das gefällt mir sehr. Freiheit“ (Adijana).

Alle Mütter berichteten von offiziellen, über die ukrainische Community organisierten oder digitalen Übersetzungs- und Dolmetschdiensten, die sie in Anspruch nahmen. Korrespondierend mit den Nachteilen der Sprachbarriere wurde der Besuch von Sprachkursen von den Müttern als sehr hilfreich angesehen. Dies lag nicht nur daran, dass Sprachkurse als notwendige Voraussetzung für eine berufliche Perspektive in Deutschland betrachtet wurden, sondern auch an dem damit verbundenen Networking und von den Lehrkräften außercurricular erbrachten Beratungsleistungen.

„Es ist gut, dass es den Sprachkurs gibt. Unser Sprachkurs ist großartig. Zumindest kommuniziere ich dort mit anderen Frauen. Wir tauschen Informationen aus, wer was weiß, wohin man gehen kann, welche Ärzte und Einrichtungen es gibt. … Die Lehrer sind so hilfreich! Ich kann der Lehrerin jede Frage stellen. In den ersten Tagen, als ich zum Arzt gehen musste, habe ich sie gefragt. Sie erklärte uns eine halbe Stunde lang, wie wir nach ihm suchen sollten“ (Milana).

Alle Interviewten hatten selbst und/oder für ihre Kinder bereits Gesundheitsdienstleistungen in Anspruch genommen. Einige berichteten von Irritationen aufgrund der Unterschiede zwischen den deutschen und ukrainischen Systemen.

„Das ist ein weiterer Punkt, warum es für die Ukrainer so schwierig ist … es ist ein anderes Medizinsystem. Wenn du in der Ukraine krank bist, geben sie dir alles und geben dir eine intravenöse Infusion. … Nun, hier gibt es einen anderen Ansatz, zumindest in der Medizin. … Er hat dir etwas verschrieben oder dich beraten oder beruhigt, aber nichts verschrieben. Nur Nurofen. Und es war sehr ärgerlich, wenn man ein krankes Kind hat und man geht hin und sie sagen: ‚Das ist Nesselsucht, macht nichts, das geht vorbei‘“ (Ana).

#### Gründe für Nichtinanspruchnahme von Angeboten.

Das Fehlen einer Betreuungsmöglichkeit für das Kind oder die Kinder war in vielen Fällen ausschlaggebend für die Nichtinanspruchnahme weiterer Angebote. Dies schilderten die Mütter vor allem im Hinblick auf ihre eigene Teilnahme an Sprachkursen (die vielfach als notwendig für eine berufliche Perspektive in Deutschland angesehen wurden). In einigen Fällen war das Kind noch zu jung für eine Fremdbetreuung oder der Kita-Besuch war bereits geplant. Dagegen wurde seltener von einem Mangel oder einer erschwerten Zugänglichkeit von Betreuungsangeboten gesprochen. Diese Ergebnisse weisen auf eine zunächst weniger stark ausgeprägte Bereitschaft der Geflüchteten hin, eine U3-Kita-Betreuung in Anspruch zu nehmen. Dies entspricht den im Vergleich zum Durchschnitt der deutschen Wohnbevölkerung geringeren Inanspruchnahmequoten bei Familien mit Fluchterfahrung [19]. Besonders schwierig war die Situation für die ohne Partner oder andere Familienmitglieder geflohenen Mütter. In einigen Fällen waren die Mütter nicht über Unterstützungsangebote informiert. Meist war der Mangel dabei nicht in der Unzugänglichkeit der Informationen begründet, sondern zumindest auch von fehlender Motivation, Zeitmangel und anderen Prioritäten beeinflusst. Es gab zudem innere Widerstände, die von der Inanspruchnahme von Unterstützungsangeboten abhalten konnten. Hier waren ein besonderer Eigenständigkeitsanspruch, wie hier formuliert: „Keiner passt auf meine Kinder auf. Das ist die Art Mensch, die ich bin“ (Marija), Scham bei der Bitte um Hilfe: „Es ist so schwer, jemanden etwas zu fragen“ (Elizaveta), und Misstrauen gegenüber Hilfeangeboten von Bedeutung:

„Ich habe einen Link zu einer Hilfeorganisation erhalten. Es heißt: ‚Wir helfen, wir haben kostenlose Babysitter, wenn Sie irgendwo hingehen müssen‘, aber ich habe kein Vertrauen. Wenn es umsonst ist, kann ich nicht der Chef sein und die Regeln diktieren“ (Adijana).

## Diskussion

In 17 Interviews von jeweils etwa einer Stunde Dauer wurden im Rahmen unseres Forschungsprojektes 17 aus der Ukraine nach Deutschland geflohene Eltern (16 Mütter, ein Vater) mit Kindern bis zu 3 Jahren zu ihrer Situation befragt. Im Rahmen der vorliegenden Analyse wurden die Belastungen und Ressourcen der Interviewten sowie ihre Kenntnis und Nutzung von Angeboten für Eltern mit kleinen Kindern exploriert.

Bei den Belastungen dominierten die Erfahrungen aus dem Krieg, der Flucht und der Besetzung. Die Interviewten berichteten von großer Angst, vor allem um das Wohlergehen ihrer Kinder. Die kriegsbedingten Belastungen waren über die Sorge um den in der Ukraine verbliebenen Partner und weitere Bezugspersonen weiterhin präsent. Bei einigen Müttern zeigten sich Anzeichen depressiver Verstimmungen wie Erschöpfung, sozialer Rückzug und Antriebslosigkeit, was dem erhöhten Anteil depressiver Symptome bei ukrainischen Geflüchteten, insbesondere bei geflüchteten Frauen, in quantitativen Untersuchungen entspricht [20, 21]. Wie in einer repräsentativen Befragung ukrainischer Geflüchteter in Deutschland [2] war Einsamkeit besonders bei den ohne Partner oder Verwandte lebenden Müttern eine Belastung. Sie könnte durch die Beschränkung der sozialen Interaktion auf den Mutter-Kind-Kontakt mit verursacht oder verstärkt werden; internationale Studien zu geflüchteten Müttern mit kleinen Kindern [22], aber auch zu Alleinerziehenden im Allgemeinen [23] deuten in die gleiche Richtung. Das tägliche Leben in Deutschland empfanden die Mütter vor allem durch die Bürokratie und mangelnde Digitalisierung in relevanten Bereichen (Ämter, Gesundheitswesen) erschwert. Eine große Hürde stellte die deutsche Sprache dar, die keine der befragten Personen vor der Flucht beherrschte, was den geringen Deutschkenntnissen gerade bei geflüchteten ukrainischen Eltern kleiner Kinder insgesamt entspricht [2]. Geringe Deutschkenntnisse erschwerten auch das Knüpfen neuer Kontakte und verstärkten Gefühle der Einsamkeit.

Bei den Ressourcen stachen die bereits bestehenden oder sich im Laufe der ersten Monate aufspannenden Netzwerke ukrainisch- und russischsprachiger Personen hervor, wie sie auch in aktueller Forschung als maßgebliche Unterstützungselemente der Ukrainerinnen und Ukrainer in Deutschland ermittelt wurden [2]. Diese Netzwerke waren oft vor allem bei alltagspraktischen Fragen von Nutzen und erfüllten damit Funktionen informationeller und instrumenteller Unterstützung [9]. Sie konnten formelle oder informelle, digitale oder analoge Organisationsformen annehmen und halfen bei Registrierung, Sprachproblemen, Wohnungssuche, Freizeit und Kinderbetreuung sowie der Vermittlung medizinischer Betreuung. Die Geflüchteten konnten hier sowohl als Hilfeempfangende wie auch als Helfende Mitglied sein und Selbstwirksamkeitserfahrungen erleben, was die Bedeutsamkeit sozialer Unterstützung für das seelische Wohlbefinden der Interviewten unterstreicht [9]. Für einen Teil der Interviewten waren die mit ihnen eingereisten Partner und/oder Familienmitglieder eine große Entlastung, insbesondere bei der zentralen Aufgabe der Kinderbetreuung. Mit Angehörigen eingereiste Personen berichteten folglich auch seltener, sich auf die Mutterrolle und den Mutter-Kind-Kontakt beschränkt zu fühlen. Als dritte unterstützende Personengruppe wurden mit großer Dankbarkeit Einzelpersonen, Initiativen und Institutionen in Deutschland erwähnt, deren Hilfe teils Erstaunen auslöste: „Deutschland ist kein Deutschland, das ist ein Wunderland“ (Viktorija). Neben diesen äußerlichen Ressourcen traten bei einigen Müttern auch psychologische Stärken hervor. Diese bestanden in den Fähigkeiten, Stressoren lösungsorientiert anzugehen, etwa beim Spracherwerb, und positive Entwicklungen und Verbesserungen wahrzunehmen, was sich beides auch in Untersuchungen zu ukrainischen Geflüchteten in Polen als vorteilhaft erwiesen hat [24, 25].

Zur Nutzung von Angeboten ist zu konstatieren, dass die Mütter sich vor allem über elektronische Medien informierten, über die die ukrainisch- und russischsprachige Community Informationen austauscht oder selbst Beratungsangebote, z. B. durch Hebammen, organisiert. Offizielle Informationsquellen, wie Webseiten der Kommunen, wurden ebenfalls frequentiert. Als Anbieter und auch als Vermittler von Unterstützungsangeboten traten Nichtregierungsorganisationen und – ähnlich wie in anderen europäischen Ländern [26] – ukrainische Kirchengemeinden auf. Die Kinderbetreuung stand bei den genutzten Angeboten stark im Vordergrund und wurde, trotz gewöhnungsbedürftiger Unterschiede zum Vorgehen in der Ukraine, als sehr positiv empfunden. Ihre Bedeutung für die sprachliche und berufliche Integration gerade der ukrainischen Mütter in Deutschland wird auch in einer aktuellen Publikation auf Basis repräsentativer Daten unterstrichen [27]. Obschon diejenigen Mütter, deren Kinder noch nicht fremdbetreut wurden, sich von einer Betreuung vor allem Entlastung für sich selbst erhofften, sprachen die Interviewten mit tatsächlich in Kitas betreuten Kindern vor allem von den positiven Auswirkungen des Kita-Besuchs auf die Kinder und deren Entwicklung. Ebenso positiv erwähnt wurden offizielle wie selbstorganisierte Sprachkurse sowie Übersetzungsdienstleistungen, die über z. B. im Gesundheitswesen zur Verfügung gestellte Dolmetscherinnen und Dolmetscher, private, zweisprachige Helfende oder digitale Dienste angeboten wurden.

Bei der Nutzung von Gesundheitsdienstleistungen (in erster Linie für die Kinder) gab es Irritationen aufgrund ungewohnter Vorgehensweisen, was die Teilnehmenden auf Unterschiede zwischen Deutschland und der Ukraine zurückführten, die auch in der aktuellen Literatur bereits beschrieben wurden [28]. Größtes Hindernis für die Inanspruchnahme weiterer Angebote war die fehlende Kinderbetreuung, die aber nicht nur einem systemischen Mangel zugeschrieben wurde, sondern auch der jeweiligen Situation der Familien: In einigen Fällen war die Betreuung in einer Kita bereits geplant oder vereinbart, hatte aber noch nicht begonnen, weil das Kind noch zu jung oder die Familie erst kürzlich in den jeweiligen Stadtteil gezogen war. Mitunter war auch das Fehlen von Informationen ein Grund für die Nichtinanspruchnahme, was oft die Folge anderer Faktoren, wie z. B. fehlender Motivation, sich zu informieren, war. Innere Widerstände wie ein starker Eigenständigkeitsanspruch, Scham oder Misstrauen konnten ebenfalls die Inanspruchnahme von Angeboten behindern. Bezogen auf das Modell der sozialen Unterstützung ist interessant, dass vor allem die instrumentelle und informationelle Unterstützung von den Interviewten erwähnt wurde, emotionale Unterstützung dagegen kaum oder eher implizit. Möglicherweise ist dies der Ankunftssituation geschuldet, in der praktische und organisatorische Fragen im Vordergrund stehen. Einschränkend muss dabei erwähnt werden, dass auch praktische Angebote als emotional unterstützend empfunden werden können, wie die große Dankbarkeit der Mütter gegenüber hilfsbereiten Deutschen andeutet.

### Limitationen.

Generell streben qualitative Vorhaben wie dieses nicht nach Repräsentativität, was die Übertragbarkeit der Ergebnisse stark einschränkt. So sind Geflüchtete aus der Ukraine gegenüber anderen Geflüchteten aufenthalts- und sozialrechtlich privilegiert [3, 4]. Darüber hinaus handelte es sich bei den Interviewten mit einer Ausnahme um Ukrainerinnen und Ukrainer in Berlin; die Perspektive von Geflüchteten, die in anderen Städten oder im ländlichen Raum leben, ist hier nicht berücksichtigt. Zudem unterscheiden sich die Bundesländer teils erheblich in Bezug auf die Unterbringung von Geflüchteten [29], die Bedarfsdeckung der Kinderbetreuung [30] und deren Kosten [31]. Die Interviews werfen ein Schlaglicht auf eine Gruppe, die sich in einem Zwischenzustand befand, teilweise noch keine längerfristige Perspektive entwickelt hatte und sich zum Zeitpunkt der Veröffentlichung bereits in einer anderen Lebenssituation befinden kann.

## Fazit

Bei den Schilderungen der Interviewten stellten sich 4 zentrale Punkte heraus: 1. die enorme psychische Belastung der Geflüchteten durch Krieg und Flucht; 2. die zentrale Bedeutung einer Kinderbetreuung für die Integration und das seelische Wohlbefinden insbesondere der ohne erwachsene Begleitung eingereisten Mütter; 3. die Wichtigkeit des Spracherwerbs für die Bewältigung des Lebens in Deutschland, inklusive der Überwindung bürokratischer Hürden und des Gelingens sozialer und beruflicher Integration; 4. die überragende Rolle selbstorganisierter Netzwerke für die Geflüchteten.

Die Ergebnisse dieser Studie, insbesondere die große psychische Belastung und potenzielle Traumatisierung aufgrund von Krieg und Flucht, sowie die häufige Erwähnung negativer Emotionen, wie Niedergeschlagenheit und Antriebslosigkeit, deuten auf einen erheblichen zukünftigen Bedarf an psychotherapeutischer Betreuung und Therapie. Dies wird sich möglicherweise verstärken, wenn die seelischen Folgen der Belastungen durch Krieg und Flucht bei den Geflüchteten noch deutlicher sichtbar werden, was mit Verzögerung geschehen kann. Dies trifft auch für Kleinkinder zu [32], weshalb Kinderbetreuungseinrichtungen für den Umgang mit potenziell traumatisierten Kindern aus Krisengebieten sensibilisiert und ausgestattet werden sollten.

Eine wichtige Entlastung würde die Bereitstellung ausreichender Kinderbetreuungsplätze mit sich bringen, die den Eltern auch den Besuch von Sprachkursen und damit eine schnellere Integration erleichtern könnte. Für die Ansprache und Einbindung der Ukrainerinnen und Ukrainer besteht mit den selbstorganisierten Netzwerken der ukrainisch- und russischsprachigen Community eine wirkmächtige Ressource, die bei der Entwicklung von Forschungsvorhaben und Interventionen für diese Zielgruppe von Beginn an partizipativ eingebunden und mit öffentlichen Mitteln unterstützt werden sollte.

## Footnotes

1 https://www.house-of-research.de/.
